# The Inferior Vena Cava Collapsibility Index as a Non-Invasive Haemodynamic Tool in Mechanically Ventilated Surgical Neonates: A Monocentric Observational Study

**DOI:** 10.3390/children13091238

**Published:** 2026-09-12

**Authors:** Carmine Mattia, Roberta Leonardi, Chiara Distefano, Maria Grazia Scuderi, Vincenzo Di Benedetto, Martino Ruggieri, Pietro Sciacca, Pasqua Betta

**Affiliations:** 1 Neonatal Intensive Care Unit, AOU Policlinico G. Rodolico San Marco, 95123 Catania, Italy; lorettamattia@hotmail.com (C.M.); mlbetta@yahoo.it (P.B.); 2 Postgraduate Training Programme in Pediatrics, Department of Clinical and Experimental Medicine, University of Catania, 95123 Catania, Italy; 3 Unit of Pediatric Clinic, Department of Clinical and Experimental Medicine, University of Catania, 95123 Catania, Italy; 4 Neonatal Intensive Care Unit, Ospedale Umberto I Siracusa, 96100 Siracusa, Italy; chiara.distefano4993@gmail.com; 5 Pediatric Surgery Unit, Department of Medical and Surgical Sciences and Advanced Technologies, G. F. Ingrassia, AOU Policlinico G. Rodolico San Marco, University of Catania, 95123 Catania, Italy; mgscuderi@gmail.com (M.G.S.); vdbchirurgiapediatrica@gmail.com (V.D.B.); 6 Unit of Pediatric Cardiology, University Hospital Policlinico, 95123 Catania, Italy; pietrosciacca@hotmail.it

**Keywords:** inferior vena cava collapsibility index, IVCCI, neonatal surgery, hypovolaemia, neonatal haemodynamics, echocardiography, cardiac output, stroke volume, mechanical ventilation, neonatal intensive care

## Abstract

**Highlights:**

**What are the main findings?**

- The IVCCI showed significant inverse correlations with cardiac output, stroke volume, peak Doppler velocities, and mean arterial pressure in critically ill mechanically ventilated surgical neonates (Spearman ρ ranging from −0.955 to −0.984; all *p* < 0.001).
- Following colloid administration (10–20 mL/kg), the IVCCI decreased significantly from 44.05 ± 7.8% to 15.14 ± 4.3% (*p* < 0.001), in parallel with significant improvements in all echocardiographic haemodynamic indices and mean arterial pressure.

**What are the implications of the main findings?**

- The IVCCI may represent a feasible, non-invasive adjunct tool for bedside haemodynamic assessment in postoperative surgical neonates on positive-pressure ventilation; however, given the exploratory design and methodological limitations, it should not be used as a standalone parameter for guiding fluid resuscitation.
- Larger prospective multicentre studies are needed to validate neonatal-specific IVCCI thresholds through ROC analysis, assess inter- and intra-observer reproducibility, and confirm the role of the IVCCI within a multiparametric haemodynamic monitoring framework.

**Abstract:**

**Background/Objectives**: Hypovolaemia and hypotension in the postoperative neonatal period are life-threatening conditions difficult to diagnose with standard clinical parameters. The inferior vena cava collapsibility index (IVCCI) is a validated non-invasive tool in adults; its application in mechanically ventilated surgical neonates remains poorly defined. This study aimed to evaluate the association of the IVCCI with echocardiographic haemodynamic indices and its changes following volume expansion in surgically treated neonates. **Methods**: This monocentric observational study enrolled 36 surgical neonates (mean gestational age 38.1 weeks; mean weight 2799 g) with postoperative hypovolaemia and oliguria at the UOC NICU-Neonatology, AOU Policlinico G. Rodolico–San Marco, Catania (July 2020–September 2023). All patients were on controlled invasive mechanical ventilation. The IVCCI, cardiac output (CO), stroke volume (SV), and peak Doppler velocities (Vmax) of the pulmonary artery and aorta were measured before and 4–6 h after colloid infusion (10–20 mL/kg). **Results**: The IVCCI decreased significantly from 44.05 ± 7.8% to 15.14 ± 4.3% (*p* < 0.001). MAP improved from 36.89 ± 5.12 to 55.99 ± 4.87 mmHg (*p* < 0.001) and pH from 7.32 ± 0.04 to 7.36 ± 0.03 (*p* < 0.01). The Vmax, SV, and CO of both ventricles increased significantly (all *p* < 0.001). Significant inverse correlations were found between the IVCCI and MAP, Vmax, CO, and SV (all *p* < 0.001). No correlation was found with gestational age, birth weight, or pain scores. **Conclusions**: The IVCCI is a feasible and clinically useful non-invasive echocardiographic parameter for haemodynamic monitoring in critically ill surgical neonates. Its significant correlation with cardiac function indices supports its potential role in the bedside assessment of suspected hypovolaemia. Larger prospective studies are warranted.

## 1. Introduction

One of the main challenges of neonatal intensive care is the early diagnosis and management of hypovolaemia/hypotension in newborns with compromised cardiorespiratory conditions.

Major surgical interventions in the first days of life frequently cause hypotension or hypovolaemia resulting in oliguria and anuria. Some clinical parameters, such as heart rate and blood pressure, must be correctly interpreted for the purposes of correct therapy so as to reliably predict the improvement in perfusion in various organs and tissues. However, these parameters have limited diagnostic usefulness, especially in newborns at risk [1], and obtaining an early diagnosis of any hypovolaemic state could help to not only improve the interpretation of these parameters but also avoid long-term neurological development consequences, which are possible complications in the case of delayed diagnosis [2].

Considering that cardiac output is determined by the amount of blood that returns to the heart (preload), the force of contractility and the resistance against which the heart must pump (afterload), it becomes very important in newborns, especially after complex surgery, to consider preload when setting treatment strategies to improve cardiovascular performance. The methods used for this evaluation are very limited in newborns [3]. Certainly, CVP is a good indicator of right ventricular function [4], but its continuous monitoring through a catheter is very invasive and, at the same time, sometimes difficult. Therefore it becomes important and at the same time necessary to use reliable and less invasive alternatives. However, the clinical applicability of the IVCCI is limited by various confounding factors, including intra-abdominal pressure, the compliance of the IVC wall, right ventricular function, and intrathoracic pressure. Current neonatal targeted echocardiography guidelines do not support the IVCCI as a standalone measure due to these limitations, emphasising the need for additional rigorous studies to clarify its diagnostic value.

Therefore, we increasingly resort to the use of echocardiographic parameters such as the end-diastolic diameter of the left ventricle, the diameter of the left atrium, and the LA/AO ratio, and in recent years, we have started to use the inferior vena cava collapsibility index (IVCCI). The IVC is the largest vein of the low-pressure venous system, and its expansion reflects, in some way, variations in venous pressure and excess intravascular volume [5], so much so that it can become an important tool for the evaluation of hypovolaemia and hypervolaemia [6].

The collapsibility index of the inferior vena cava (IVCCI) is now constantly used in clinical practice in the adult population [7] as a predictive factor of hypovolaemic shock. This index is defined as the difference between the maximum expiratory diameter and the minimum inspiratory diameter divided by the maximum expiratory diameter, which, in the literature, is reported as pathological and therefore an indicator of hypovolaemia if >40% [8].

Prior studies have evaluated IVC collapsibility or distensibility in neonates and paediatric patients in various clinical contexts, including fluid resuscitation, sepsis, and haemodialysis [6,9,10]. However, to our knowledge, no previous study has specifically assessed the IVC collapsibility index in mechanically ventilated surgical neonates during the postoperative period and in relation to postoperative fluid resuscitation. We therefore consider this the primary novelty of our work. We also acknowledge that, in mechanically ventilated patients, the term IVC distensibility index (IVCD) is physiologically more accurate than IVC collapsibility index (IVCCI), as positive-pressure ventilation induces IVC distension rather than collapse during the inspiratory phase. Nevertheless, we retained the term IVCCI throughout this manuscript to maintain consistency with the majority of the published literature and with the formula used [(IVC_max_ − IVC_min_)/IVC_max_ × 100%], acknowledging that this terminology represents a recognised limitation in the context of mechanical ventilation.

Given this evidence gap, this study aimed to correlate the IVCCI with cardiac output (CO) and stroke volume (SV) before and after colloid administration in surgical neonates presenting with postoperative hypotension and oliguria and to assess its potential as a timely, non-invasive indicator of clinically significant hypovolaemia.

## 2. Materials and Methods

This is a retrospective monocentric observational study conducted at the UOC NICU-Neonatology of the AOU Policlinico G. Rodolico–San Marco, Catania, Italy, between July 2020 and September 2023. Written informed consent was obtained from all parents or legal guardians prior to enrolment.

Hypovolaemia was defined as a clinical condition characterised by oliguria (<1 mL/kg/h) in association with at least one of the following: heart rate > 180 beats/min or decreased systemic blood pressure below age-appropriate norms. This composite definition was chosen to reflect the multifactorial nature of postoperative hypovolaemia in the neonatal setting, where no single parameter is sufficiently specific as a standalone criterion.

### 2.1. Echocardiographic Assessment and IVCCI Measurement

Echocardiographic examinations were performed with a Philips EPIQ 7 ultrasound system (Philips Healthcare, Amsterdam, The Netherlands) equipped with an S8-3 sector transducer (frequency range 3–8 MHz) by operators trained in neonatal functional echocardiography. All examinations were performed with the neonate in the supine position during a period of clinical stability, with sedoanalgesia maintained as described above. IVC measurements were obtained via the subcostal longitudinal view, with the probe positioned at the subxiphoid level and rotated 90° counterclockwise to visualise the IVC entering the right atrium. M-mode was placed 1–2 cm caudal to the IVC–right atrial junction, with a sweep speed of 50–100 mm/s. The maximum (expiratory) and minimum (inspiratory) IVC anteroposterior diameters were measured over three to five consecutive respiratory cycles and averaged. In mechanically ventilated patients, measurements were synchronised with the ventilator cycle. The IVCCI was calculated as [(IVC_max_ − IVC_min_)/IVC_max_] × 100%, where IVC_max_ is the maximum expiratory diameter and IVC_min_ the minimum inspiratory diameter. The cardiac output (CO) and stroke volume (SV) of the right and left ventricles were calculated from the outflow tract diameters (RVOT, LVOT) and velocity–time integrals (VTIs) obtained by pulsed-wave Doppler: SV = (π × (d/2)^2^) × VTI; CO = SV × HR. Peak Doppler flow velocities (Vmax) were measured in the main pulmonary artery and ascending aorta. All measurements were performed by the same operator and averaged over three cardiac cycles. Inter- and intra-observer variability were not formally assessed, which represents a limitation of this study.

### 2.2. Statistical Analysis

Statistical analysis was performed using Microsoft Excel (Microsoft Corporation, Redmond, WA, USA). The normality of continuous variables was assessed using the Shapiro–Wilk test. As most variables did not follow a normal distribution, nonparametric tests were used throughout: correlations between the IVCCI and haemodynamic parameters were assessed using the Spearman rank correlation coefficient (ρ), and paired pre–post comparisons were performed using the Wilcoxon signed-rank test. The results are reported as medians and interquartile ranges (IQRs) for non-normally distributed variables and as means ± SD for normally distributed ones. A *p*-value < 0.05 was considered statistically significant. No formal a priori power calculation was performed; this study was exploratory in nature and enrolled all eligible patients over the study period. Multivariate analysis controlling for potential confounders was not performed and represents a further limitation of this study.

## 3. Results

Thirty-six neonates were enrolled. The baseline demographic and clinical characteristics of the study population, including surgical diagnoses, are reported in the Results Section (Table 1 and Table 2). Additional demographic data including sex distribution and postnatal age at the time of echocardiographic evaluation were not systematically recorded in the clinical database and could not be retrieved; this represents a limitation of this study. All patients were managed on controlled pressure-limited invasive mechanical ventilation and received sedoanalgesia according to pain scores (PIPP scale) with morphine (0.02 mg/kg/h) and midazolam (0.06 mg/kg/h). A plasma expander was administered at 10–20 mL/kg to all enrolled patients; none required vasopressor therapy.

The first echocardiographic assessment was performed at a mean of approximately 4 h after surgery, prior to colloid infusion. The second assessment was performed 4–6 h after the end of colloid infusion (range 4–6 h). The resumption of diuresis (defined as urine output > 1 mL/kg/h for at least 2 consecutive hours) was observed in all patients at a mean of approximately 6 h after colloid administration (approximately 8 h after surgery).

**Figure 1:**
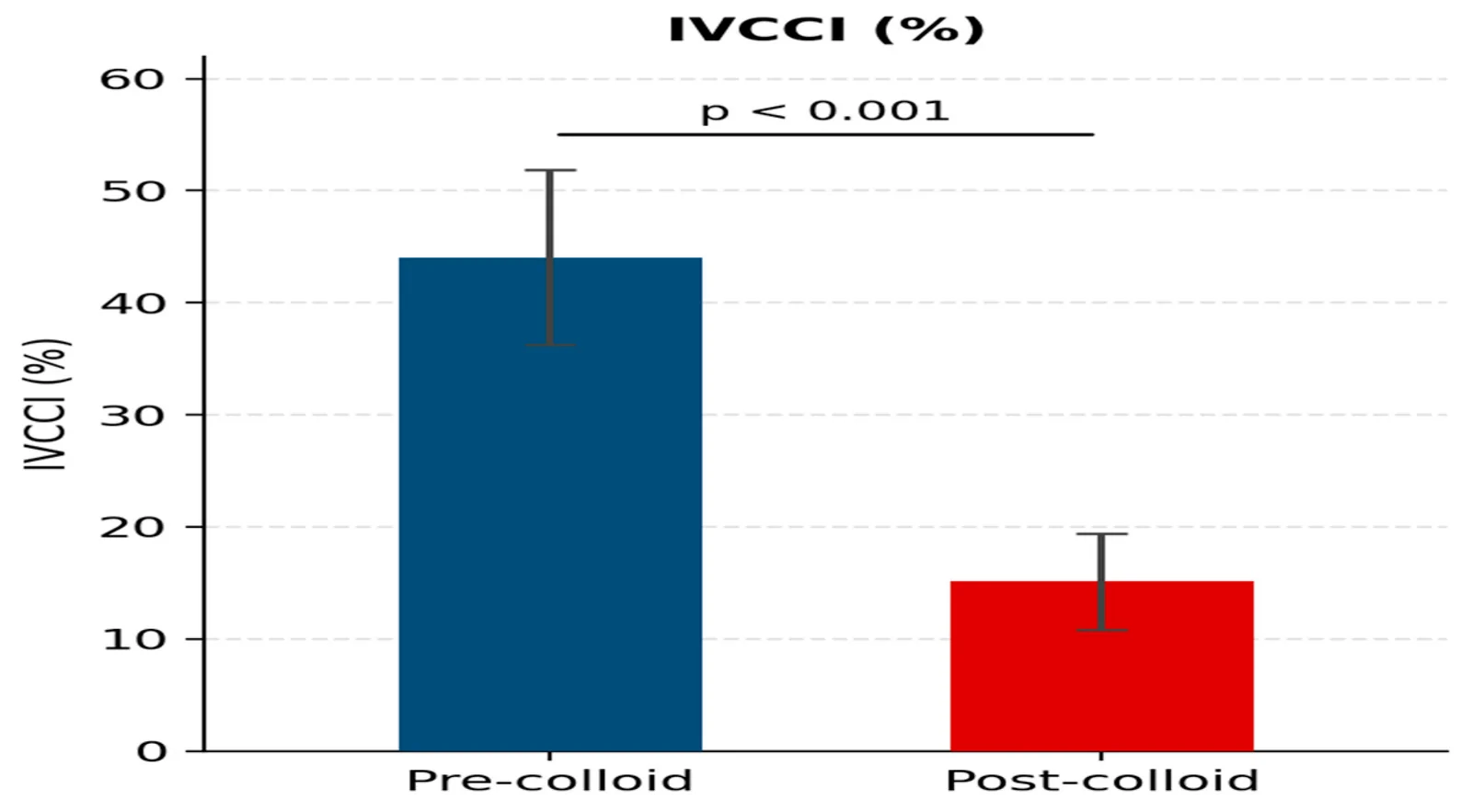
IVCCI variations before and after colloid infusion. Bars represent mean ± SD. *p* < 0.001 (Wilcoxon signed-rank test).

**Figure 2:**
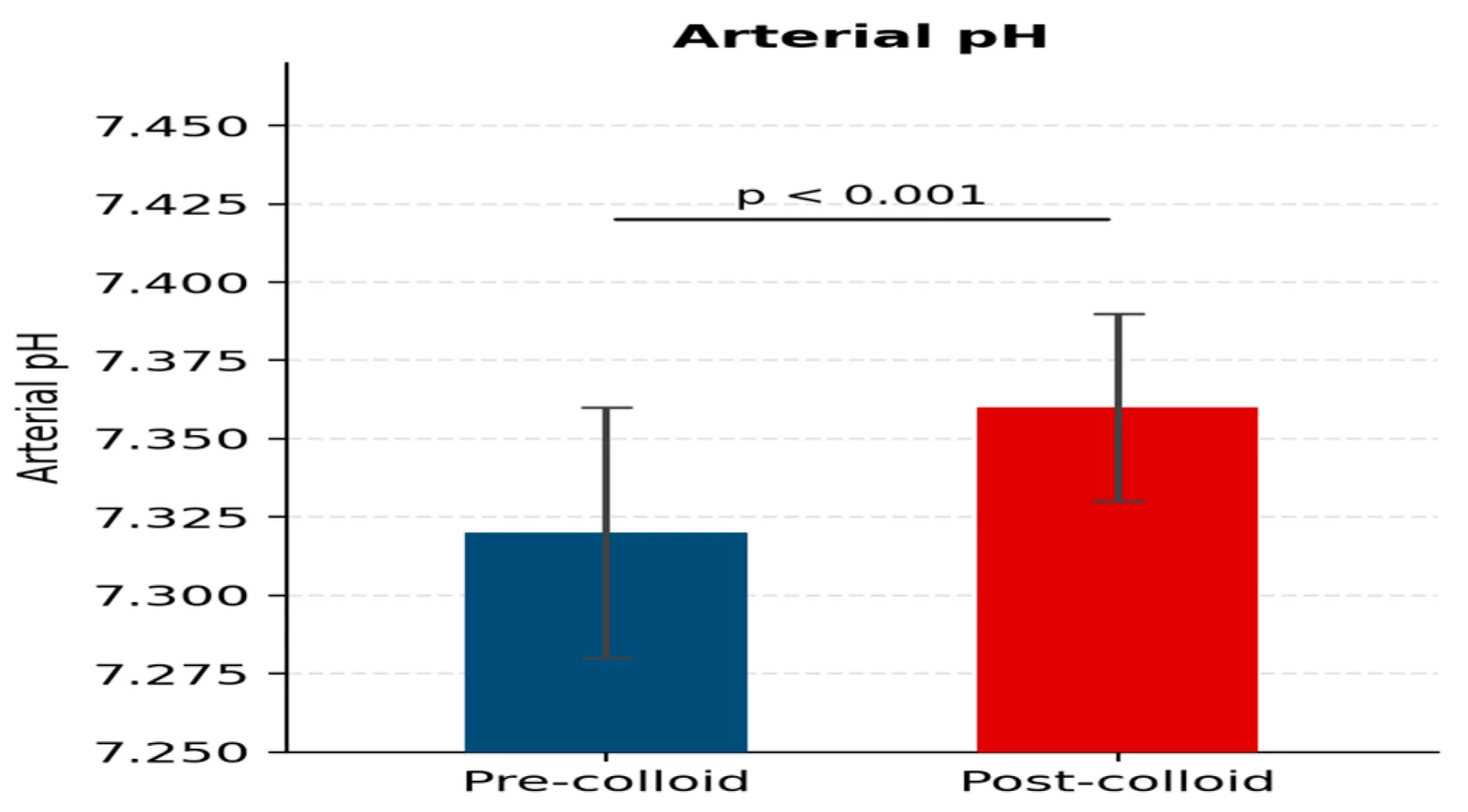
Arterial pH before and after colloid infusion. Bars represent mean ± SD. *p* < 0.001 (Wilcoxon signed-rank test).

**Figure 3:**
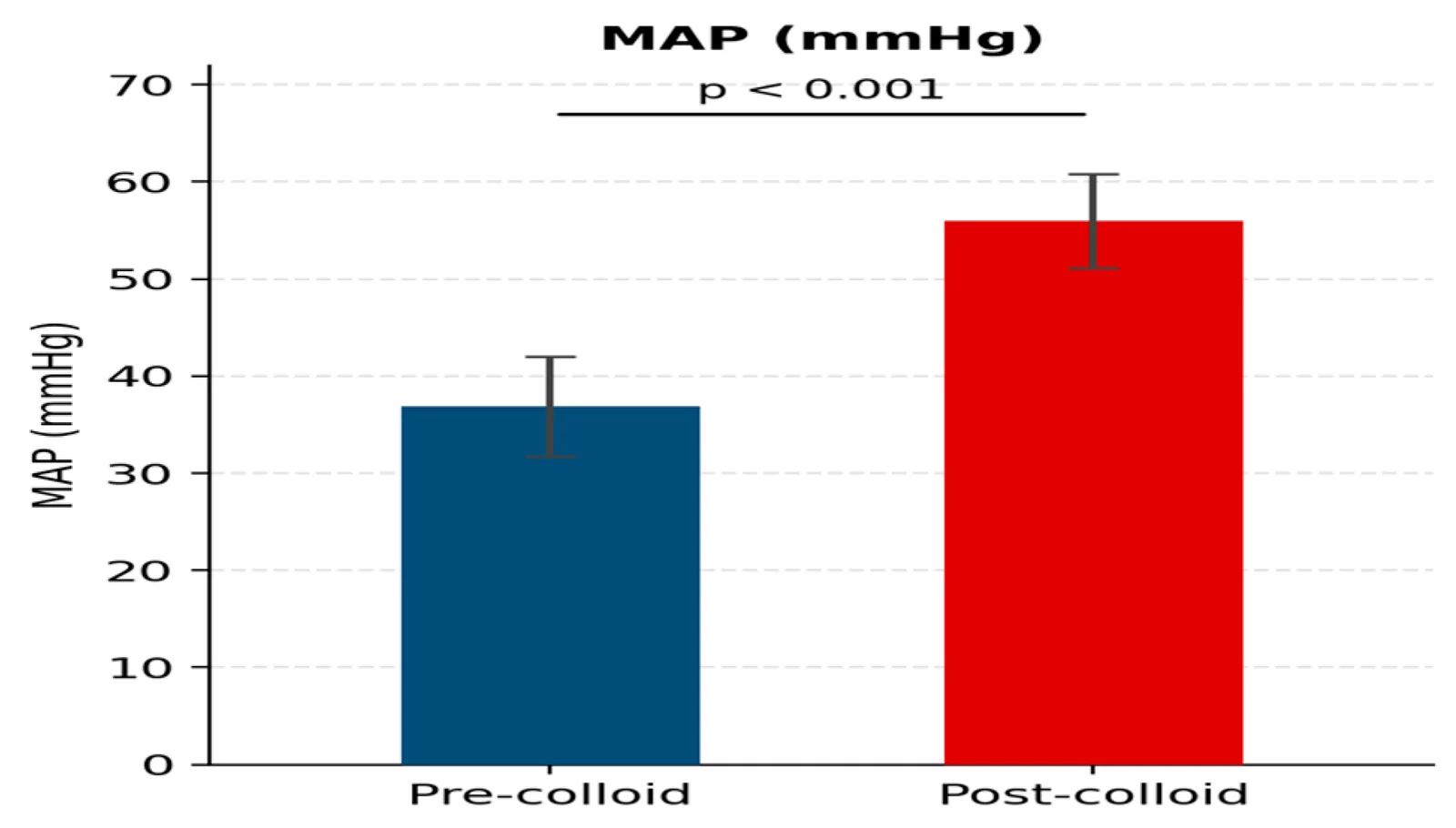
Mean arterial pressure (MAP, mmHg) before and after colloid infusion. Bars represent mean ± SD. *p* < 0.001 (Wilcoxon signed-rank test).

The IVCCI decreased significantly from 44.05 ± 7.8% to 15.14 ± 4.3% after colloid infusion (*p* < 0.001) (Figure 1), inversely proportional to improvements in cardiac function parameters. Mean pH improved significantly from 7.32 ± 0.04 to 7.36 ± 0.03 (*p* < 0.01) (Figure 2). MAP increased significantly from 36.89 ± 5.12 mmHg to 55.99 ± 4.87 mmHg (*p* < 0.001) (Figure 3).

Pulmonary artery Vmax increased from 0.70 ± 0.12 to 0.94 ± 0.11 m/s (*p* < 0.001) and aortic Vmax from 0.63 ± 0.10 to 1.01 ± 0.13 m/s (*p* < 0.001). Right ventricular SV increased from 2.75 ± 0.65 to 3.66 ± 0.60 cm^3^ (*p* < 0.001) and left ventricular SV from 2.79 ± 0.67 to 3.78 ± 0.62 cm^3^ (*p* < 0.001). Right ventricular CO increased from 0.47 ± 0.11 to 0.71 ± 0.13 L/min and left ventricular CO from 0.50 ± 0.12 to 0.73 ± 0.14 L/min (both *p* < 0.001). All values are summarised in Table 3 together with the Spearman ρ coefficients for the correlation with the IVCCI.

**Figure 4:**
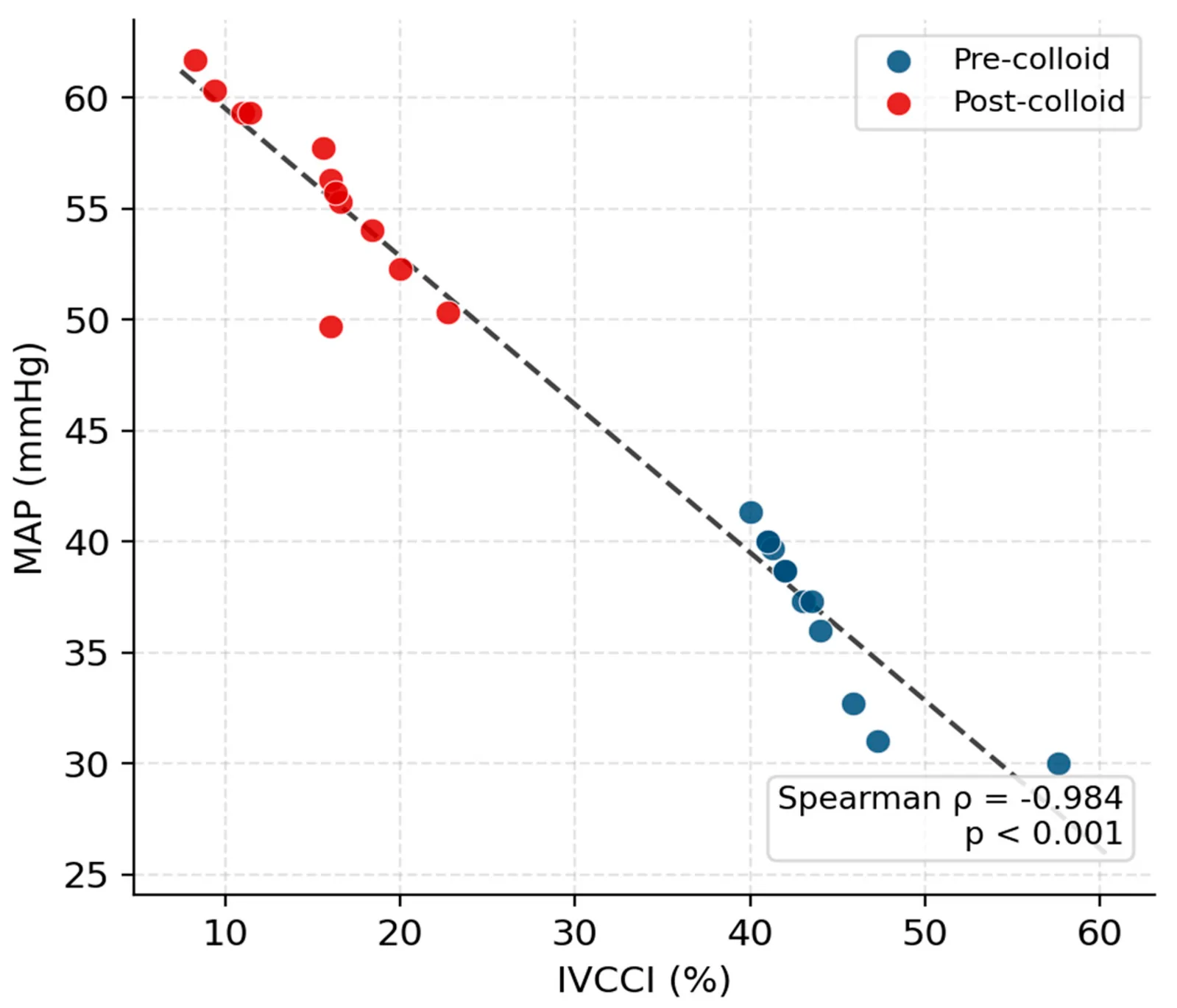
Scatter plot of IVCCI (%) vs. mean arterial pressure (MAP, mmHg). Blue: pre-colloid. Red: post-colloid. The dashed line represents the linear regression line. Spearman ρ = −0.984, *p* < 0.001.

**Figure 5:**
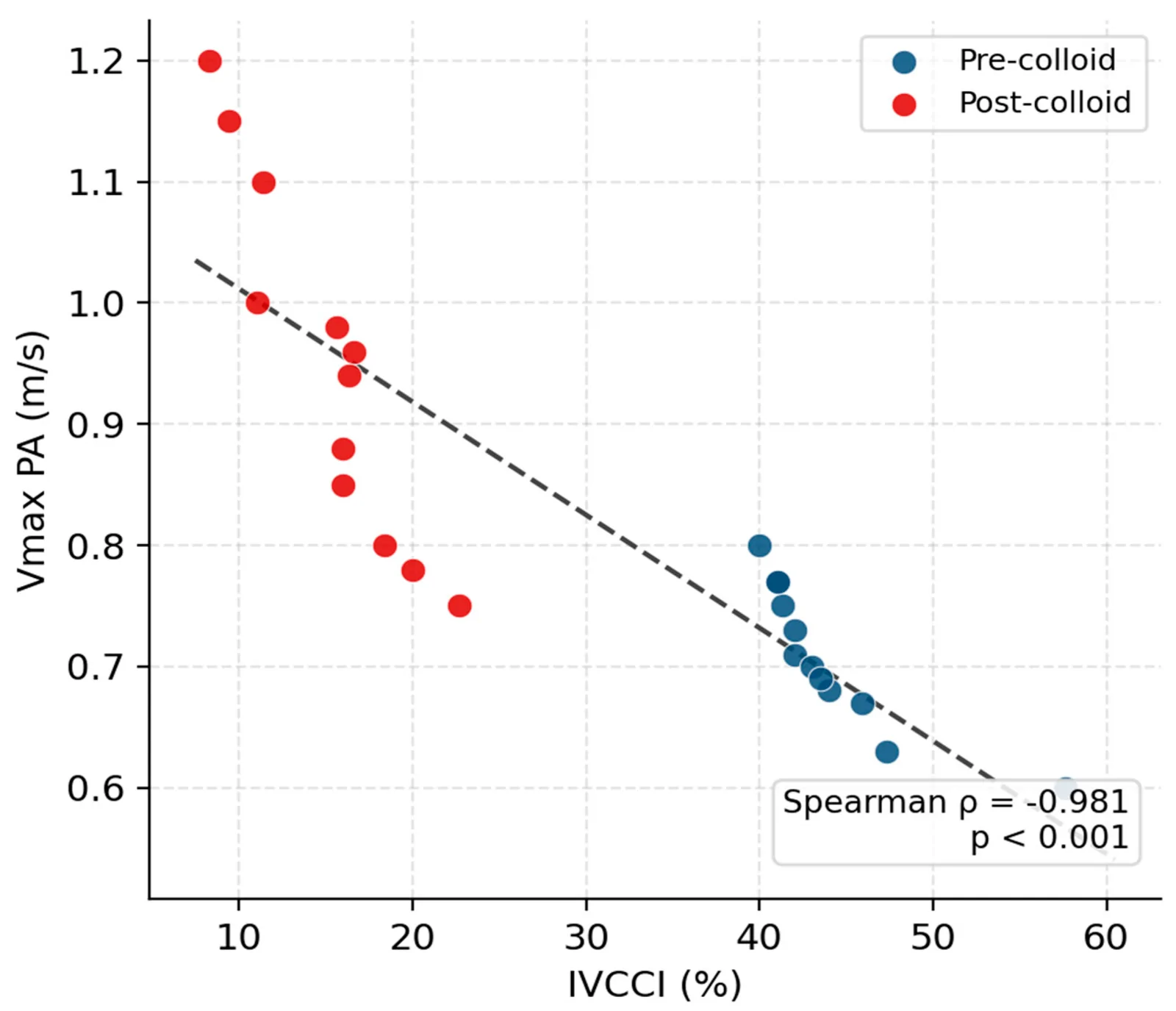
Scatter plot of IVCCI (%) vs. peak Doppler velocity in pulmonary artery (Vmax PA, m/s). The dashed line represents the linear regression line. Spearman ρ = −0.955, *p* < 0.001.

**Figure 6:**
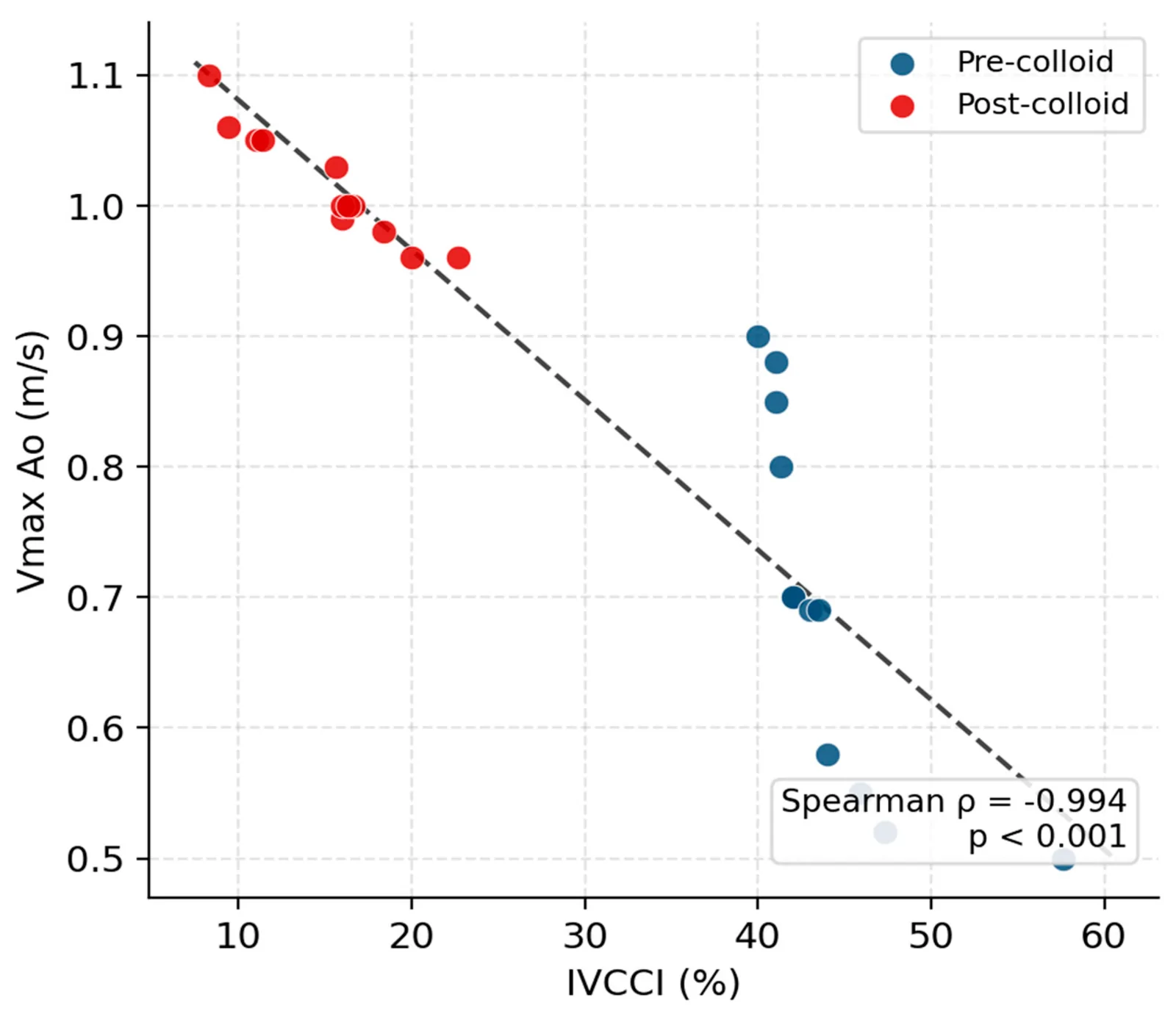
Scatter plot of IVCCI (%) vs. peak Doppler velocity in aorta (Vmax Ao, m/s). The dashed line represents the linear regression line. Spearman ρ = −0.968, *p* < 0.001.

**Figure 7:**
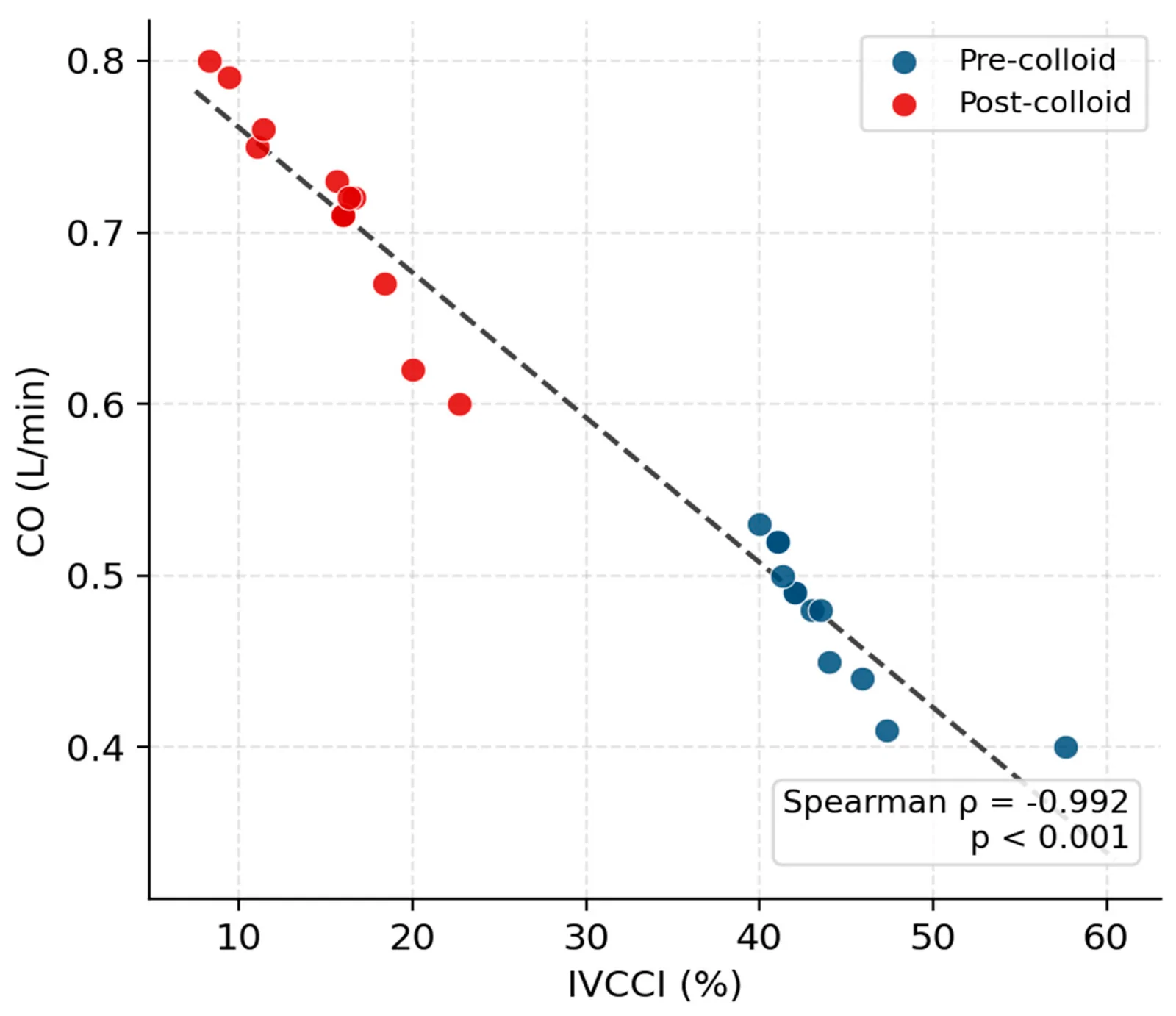
Scatter plot of IVCCI (%) vs. cardiac output (CO, L/min). Blue: pre-colloid. Red: post-colloid. The dashed line represents the linear regression line. Spearman ρ = −0.967 (RV), −0.979 (LV), *p* < 0.001.

**Figure 8:**
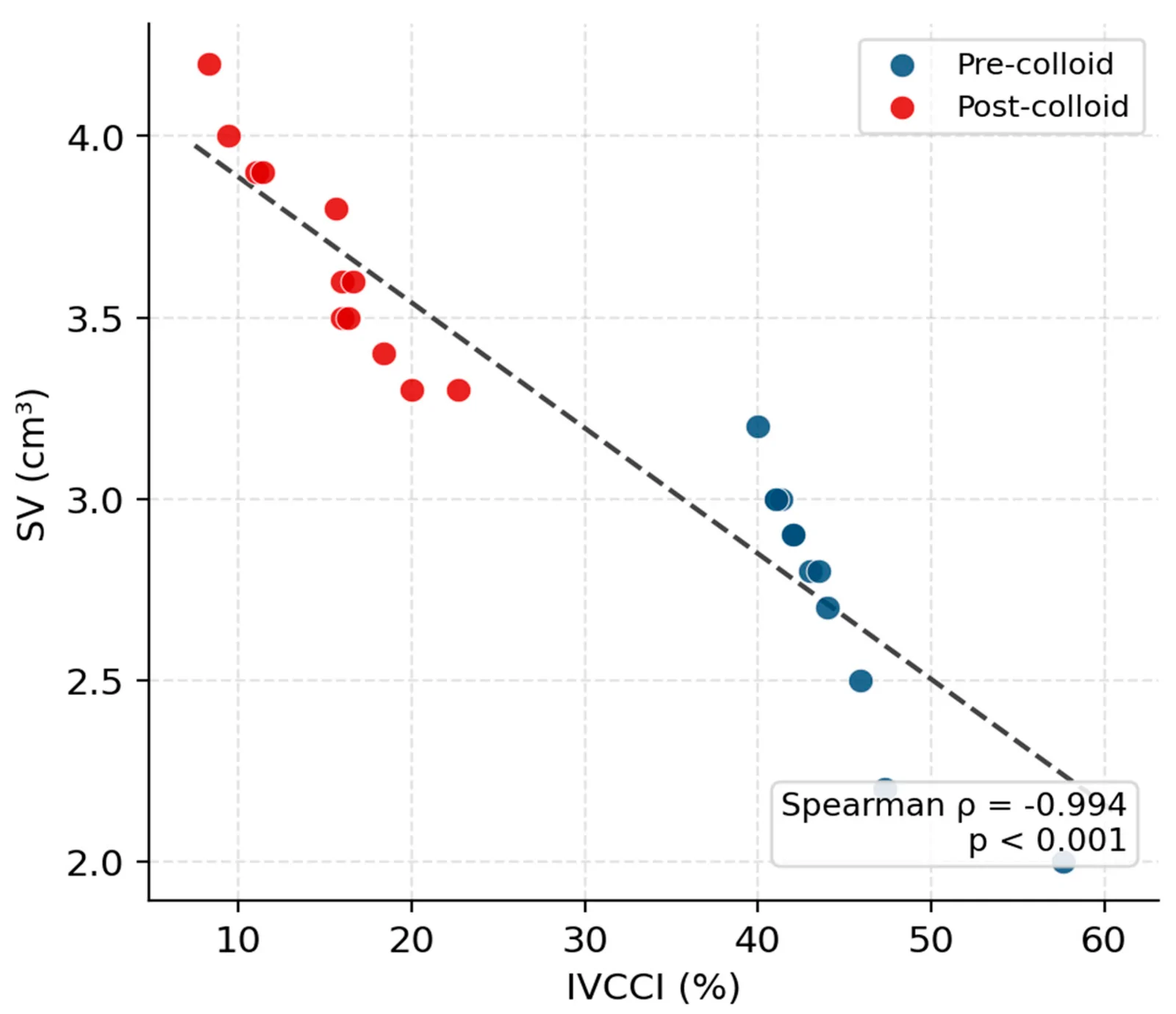
Scatter plot of IVCCI (%) vs. stroke volume (SV, cm^3^). Blue: pre-colloid. Red: post-colloid. The dashed line represents the linear regression line. Spearman ρ = −0.979 (RV), −0.965 (LV), *p* < 0.001.

We found a significant correlation between the IVCCI and MAP (*p* < 0.001) and between the IVCCI and Vmax of the pulmonary artery and aorta (*p* < 0.001), as well as between the IVCCI and CO (*p* < 0.001) and between the IVCCI and SV (*p* < 0.001). Significant inverse correlations were observed between the IVCCI and MAP (ρ = −0.984, *p* < 0.001), between the IVCCI and Vmax of the pulmonary artery (ρ = −0.955, *p* < 0.001) and aorta (ρ = −0.968, *p* < 0.001), between the IVCCI and CO of the right ventricle (ρ = −0.967, *p* < 0.001) and left ventricle (ρ = −0.979, *p* < 0.001), and between the IVCCI and SV of the right ventricle (ρ = −0.979, *p* < 0.001) and left ventricle (ρ = −0.965, *p* < 0.001) (Figure 4, Figure 5, Figure 6, Figure 7 and Figure 8). No significant correlation was found between the IVCCI and pH.

## 4. Discussion

Hypovolaemic shock in neonates is a condition in which circulatory function is insufficient to meet the metabolic demands of tissues [11]. Volume expansion is a cornerstone of haemodynamic management in postoperative haemodynamic compromise; however, excessive fluid loading may worsen morbidity and mortality through tissue oedema and organ dysfunction [12,13]. The haemodynamic response to colloid infusion—an increase in stroke volume—relies on the Frank–Starling mechanism [14]. Standard clinical parameters, including heart rate and blood pressure, have limited reliability for guiding volume expansion in neonates, despite their value in older children and adults [15,16].

The IVCCI has been extensively validated as a predictor of fluid responsiveness and hypovolaemia in adult critically ill populations [17]. In adult mechanically ventilated patients with sepsis, Schefold et al. demonstrated that IVC diameter correlated significantly with invasive haemodynamic measurements including CVP [5], supporting its use as a surrogate of preload. However, the direct extrapolation of adult-derived IVCCI thresholds to the neonatal population requires caution. Neonates differ substantially from adults in terms of cardiovascular physiology, chest wall compliance, IVC dimensions, and the relative contribution of right ventricular afterload to overall haemodynamics. Specifically, the commonly used adult threshold of an IVCCI > 40% as an indicator of haemodynamically significant hypovolaemia [7,8] has not been prospectively validated in neonates. In our study, the mean pre-colloid IVCCI of 44.05 ± 7.8% was observed in the context of clinically defined hypovolaemia, and its decrease to 15.14 ± 4.3% following volume expansion is consistent with the restoration of adequate preload. While these findings are biologically plausible and directionally aligned with the adult literature, we acknowledge that a formal threshold validation—including receiver operating characteristic (ROC) curve analysis and the calculation of sensitivity, specificity, and area under the curve (AUC)—was not performed. The application of an adult-derived cut-off of >40% to this population therefore requires cautious interpretation, and prospective studies incorporating ROC analyses are needed before a neonatal-specific threshold can be recommended.

From a neonatal perspective, published data on the IVCCI remain limited. Sato et al. [10] demonstrated that IVC diameter provides a non-invasive estimate of CVP in neonates, establishing a physiological basis for IVC-based haemodynamic assessment in this population. Kieliszczyk et al. [9] reviewed the utility of ultrasound-based fluid status evaluation in neonates and acknowledged the potential of IVC assessment while highlighting the absence of validated measurement protocols. International POCUS guidelines for critically ill neonates and children (ESPNIC, 2020) [18] acknowledge IVC collapsibility as a tool for intravascular status evaluation but explicitly refrain from endorsing a standalone threshold, citing insufficient paediatric and neonatal evidence. Our findings are consistent with these prior observations and extend them to the specific context of postoperative surgical neonates. Notably, we did not identify a significant association between the IVCCI and gestational age or birth weight, suggesting that—within our cohort—the IVCCI was not significantly influenced by these variables. This is in agreement with Sato et al. [10] but contrasts with a subset of earlier studies reporting size-dependent IVC dimensions. The clinical relevance of this finding is that the IVCCI may be applicable across a range of gestational ages and birth weights, although confirmation in larger prospective cohorts is required.

All patients in our series were on controlled pressure-limited invasive mechanical ventilation. This is a critically important methodological consideration, as positive-pressure ventilation profoundly alters IVC respiratory dynamics compared to spontaneous breathing. During spontaneous inspiration, negative intrathoracic pressure increases venous return and reduces IVC diameter, resulting in IVC collapse. In contrast, during positive-pressure ventilation, inspiratory phases are associated with increased intrathoracic pressure, which impedes venous return and may paradoxically distend the IVC. As a consequence, in mechanically ventilated patients, the respiratory variation in IVC diameter—and hence the IVCCI—reflects a different physiological mechanism than in spontaneously breathing individuals. Luecke and Pelosi [19] demonstrated that positive end-expiratory pressure (PEEP) and tidal volume directly influence cardiac output and venous return, which in turn modify IVC dimensions. In our cohort, all neonates were managed with standardised sedoanalgesia (morphine 0.02 mg/kg/h and midazolam 0.06 mg/kg/h) and controlled ventilatory settings, which may have partially reduced inter-individual variability in intrathoracic pressure. Nevertheless, individual differences in respiratory mechanics, lung compliance, and PEEP levels—which were not systematically recorded—represent uncontrolled confounders that may have influenced IVCCI values independently of preload status. Future studies should systematically document ventilatory parameters, including PEEP, tidal volume, peak inspiratory pressure, and respiratory rate, to enable the appropriate stratification and adjustment of IVCCI measurements in mechanically ventilated neonates. Despite these limitations, the significant and consistent inverse correlations we observed between the IVCCI and all haemodynamic parameters suggest that the IVCCI retains clinical signals even in the context of positive-pressure ventilation, in agreement with Schefold et al. [5] and partially discordant with Stawicki et al. [20], who did not demonstrate a significant correlation between the IVCCI and CVP in a ventilated adult cohort.

The potential influence of sedation on the IVCCI deserves specific consideration. Opioids and benzodiazepines—used in our cohort for analgosedation—may reduce sympathetic tone and vascular resistance, potentially altering venous capacitance and IVC filling independently of true intravascular volume status. Morphine, in particular, has vasodilatory properties that could influence venous return and IVC dimensions. While the uniform sedation protocol adopted in our study limits inter-patient variability due to this confounding factor, its absolute effect on the IVCCI cannot be excluded. Future investigations should consider standardising sedation regimens or, where feasible, assessing the IVCCI at different levels of sedation depth to disentangle its independent contribution.

The heterogeneity of surgical diagnoses in our cohort—ranging from oesophageal atresia and diaphragmatic hernia to gastroschisis and anorectal malformation—introduces further potential confounders. Different surgical conditions are associated with varying degrees of fluid loss, third-spacing, inflammatory response, and postoperative haemodynamic instability, all of which may influence both the IVCCI and the haemodynamic parameters against which it was correlated. In particular, conditions involving increased intra-abdominal pressure (such as gastroschisis, omphalocele, and meconium ileus) may independently compress the IVC, artificially reducing its calibre and attenuating respiratory variation independently of intravascular volume status. This represents an important limitation that we were unable to address given the observational, uncontrolled design of this study. Future research should account for intra-abdominal pressure and stratify IVCCI performance by surgical diagnosis. Nonetheless, the consistent inverse correlations observed across all diagnostic subgroups support the hypothesis that the IVCCI may carry haemodynamic information across a range of postoperative neonatal conditions.

A further important limitation of this study is the absence of formal inter- and intra-observer variability assessment for IVCCI measurements. The IVCCI is known to be operator-dependent, and reproducibility is influenced by patient positioning, subcostal acoustic window quality, the correct identification of the IVC–right atrial junction, accurate M-mode placement, and synchronisation with the ventilator cycle. In the adult literature, reported inter-observer variability for IVC measurements ranges from 10% to 15% depending on the setting and operator experience [5,20,21]. In neonates, these challenges may be amplified by smaller anatomical structures, limited acoustic windows in postoperative patients, and the need for the simultaneous management of ventilatory support. In our study, all measurements were performed by a single trained operator, which ensures intra-patient consistency but precludes the assessment of inter-observer reproducibility. This is a recognised limitation and represents a priority for future validation studies, which should formally report both intra- and inter-observer coefficients of variation before the IVCCI can be adopted as a reliable routine monitoring tool in neonatal intensive care.

This study has several additional limitations. The sample size (*n* = 36) is small, and the absence of a control group limits causal inference and generalisability. Multivariate analysis controlling for potential confounders was not performed. Furthermore, the Spearman correlation coefficients reported were computed by pooling pre- and post-colloid observations (72 data points from 36 patients); because each patient contributes two non-independent measurements and because the intervention simultaneously shifts both the IVCCI and all haemodynamic parameters in opposite directions, this approach may have substantially inflated the observed ρ values (ranging from −0.955 to −0.984). These exceptionally high correlation coefficients should therefore be interpreted with caution, and their validity should be confirmed by an analysis restricted to pre-colloid values alone or by a mixed-effects or repeated-measures approach that appropriately accounts for the paired structure of the data. We acknowledge this as a major methodological limitation. Finally, the IVCCI is subject to additional confounders in the neonatal setting—including intra-abdominal pressure, IVC wall compliance, and right ventricular afterload—that were not systematically assessed. The inclusion of neonates with diverse surgical diagnoses provides preliminary evidence across a heterogeneous surgical population; the consistent correlations observed across different diagnoses suggest that the IVCCI may be applicable regardless of the specific underlying surgical condition, although this hypothesis requires prospective validation in adequately powered, multicentre studies.

## 5. Conclusions

Neonatal haemodynamic assessment cannot rely solely on heart rate, blood pressure, and urine output. The inferior vena cava is readily accessible to bedside ultrasound, and its respiratory variation reflects changes in venous return and intravascular volume status. In this exploratory monocentric observational study, the IVCCI showed significant inverse correlations with multiple echocardiographic haemodynamic indices—including cardiac output, stroke volume, peak Doppler velocities, and mean arterial pressure—in a cohort of critically ill surgical neonates on positive-pressure mechanical ventilation. These findings are hypothesis-generating and suggest that the IVCCI may provide useful additional information on intravascular volume status at the bedside. However, given the small sample size, the absence of a control group, the observational design, the lack of a validated neonatal-specific threshold, the absence of inter-observer reproducibility data, and the statistical limitations related to pooled correlation analysis, these results must be interpreted with considerable caution and should not be used to support the routine clinical application of the IVCCI in isolation.

The IVCCI may represent a promising complementary tool within a multiparametric neonatal functional echocardiography assessment, rather than a standalone diagnostic parameter. Its potential clinical value should be confirmed in larger, prospective, multicentre studies that formally assess inter- and intra-observer reproducibility, define neonatal-specific thresholds through ROC analysis, control for ventilatory and pharmacological confounders, and compare IVCCI performance across different surgical diagnoses and gestational age groups.

From a clinical standpoint, the current evidence does not support the use of the IVCCI as a standalone parameter for guiding postoperative fluid resuscitation in neonates. The complexity of neonatal haemodynamics, the influence of mechanical ventilation on IVC respiratory variation, the absence of a validated neonatal-specific threshold, and the lack of reproducibility data collectively preclude its independent clinical application at this stage. Rather, the IVCCI should be considered a potentially informative adjunct within a structured multiparametric haemodynamic assessment framework—integrated with conventional echocardiographic indices (left ventricular end-diastolic diameter, LA/Ao ratio, fractional shortening), clinical parameters (heart rate, blood pressure, urine output), and biochemical markers (lactate, pH)—to support—but not replace—clinical decision-making in the postoperative neonatal period.

## Figures and Tables

**Table 1:** The baseline demographic and clinical characteristics of the study population (*n* = 36).

Characteristic	Value
Gestational age, weeks (mean)	38.1
Birth weight, g (mean)	2799

**Table 2:** Surgical diagnoses and patient distribution in the observational study.

Diagnosis	Nr of Cases
Intestinal duplication	2
Oesophageal atresia	7
Intestinal atresia	3
Meconium ileus	5
Anorectal malformation	4
Diaphragmatic hernia	7
Omphalocele	3
Gastroschisis	4
CAM	1

**Table 3:** Echocardiographic and haemodynamic parameters before and after colloid administration, with Spearman ρ correlation coefficients with respect to IVCCI. Data are expressed as mean ± SD. * *p*-values refer to Wilcoxon signed-rank test (pre- vs. post-colloid). Spearman ρ and *p* (ρ) were computed by pooling pre- and post-colloid observations (*n* = 72); see limitations. PA = pulmonary artery; Ao = aorta; SV = stroke volume; CO = cardiac output; RV = right ventricle; LV = left ventricle; MAP = mean arterial pressure; IVCCI = inferior vena cava collapsibility index.

Parameter	Pre-Colloid Mean ± SD	Post-Colloid Mean ± SD	*p*-Value *	Spearman ρ	*p* (ρ)
Vmax PA (m/s)	0.70 ± 0.12	0.94 ± 0.11	<0.001	−0.955	<0.001
Vmax Ao (m/s)	0.63 ± 0.10	1.01 ± 0.13	<0.001	−0.968	<0.001
SV RV (cm^3^)	2.75 ± 0.65	3.66 ± 0.60	<0.001	−0.979	<0.001
SV LV (cm^3^)	2.79 ± 0.67	3.78 ± 0.62	<0.001	−0.965	<0.001
CO RV (L/min)	0.47 ± 0.11	0.71 ± 0.13	<0.001	−0.967	<0.001
CO LV (L/min)	0.50 ± 0.12	0.73 ± 0.14	<0.001	−0.979	<0.001
MAP (mmHg)	36.89 ± 5.12	55.99 ± 4.87	<0.001	−0.984	<0.001
IVCCI (%)	44.05 ± 7.80	15.14 ± 4.30	<0.001	—	—

## Data Availability

The original contributions presented in this study are included in the article. Further inquiries can be directed to the corresponding authors.

## Abbreviations

The following abbreviations are used in this manuscript:

CO	Cardiac Output
CVP	Central Venous Pressure
IVC	Inferior Vena Cava
IVCCI	Inferior Vena Cava Collapsibility Index
IQR	Interquartile Range
LA/AO	Left Atrium-to-Aortic Root Ratio
LV	Left Ventricle
MAP	Mean Arterial Pressure
NICU	Neonatal Intensive Care Unit
PIPP	Premature Infant Pain Profile
RV	Right Ventricle
SD	Standard Deviation
SV	Stroke Volume
Vmax	Peak Doppler Velocity/Maximum Velocity
